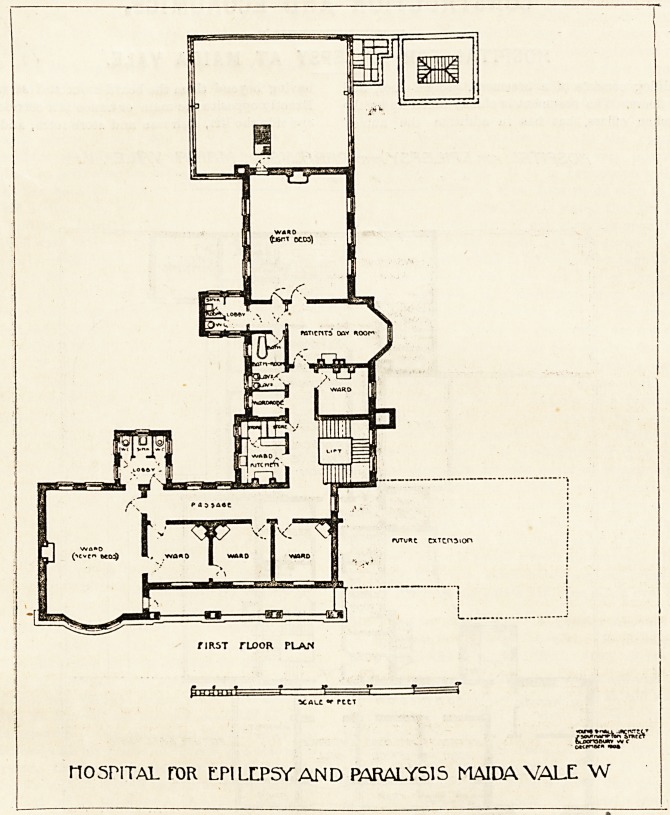# Hospital for Epilepsy at Maida Vale

**Published:** 1904-04-16

**Authors:** 


### HOSPITAL ADMINISTRATION.

### CONSTRUCTION AND ECONOMICS.

### HOSPITAL FOR EPILEPSY AT MAIDA VALE.

THIS building consists of a basement, ground floor, and three other floors. The basement is chiefly taken up by the various kitchen offices, but has in addition the nurses' dining-room and the battery-room. The ground-floor plan is at present rather onesided-looking, as so far only one wing has been built; but when the extensions shall have been put up it will be symmetrical. This floor now contains the entrance-hall, and to th left are the matron's rooms, having beyond them the board-room and secretary's office. Exactly opposite the main entrance is a corridor, having on one side the lift, staircase and store-room, and on the other side the linen-room and pantry. At the end of this corridor are the physical exercise room, dispensary, waiting-room and consulting-room. Not far from the waiting-room is the mortuary. All the component parts of this floor seem well arranged and are very compact.

HOSPITAL FOR EPILEPSY AND PARALYSIS, MAIDA VALE. W.

The first and second floors constitute the hospital proper. Over the board-room and office there is a ward for seven beds. This room has three windows at one end and two at the other, so that cross-ventilation has not been forgotten.

The lift, which has already been mentioned, is large enough to take a bed with its patient and two nurses, and it runs from basement to the top floor.

The accommodation is for 38 beds, and the cost exceeded

HOSPITAL FOR EPILEPSY AND PARALYSIS MAIDA VALE W

There is a single-bedded ward opening from the seven-bedded ward, and two other single-bedded wards adjoin it; all these have windows opening on to a broad balcony. Over the physical exercise room is another ward for eight beds. This ward is well lighted and cross-ventilated, and from it a door opens on to a lead flat, or rather a roof garden, which covers the out-patients' department. Next to the eight-bedded ward is the day-room, with a single-bedded room attached to it. The ward kitchen is conveniently placed, and so is the bath-room. The sanitary annexes are good, and they are sufficiently cut off from the main building by cross-ventilated lobbies. The second floor is similar to the first, except that it does not have the benefit of the roof garden.

The top floor is devoted to rooms for the resident staff, there being eight bedrooms for nurses, a large sitting-room opening on to a balcony for the nurses' use, a bedroom for the matron, and five bedrooms for the domestic servants.

£25,000. The hospital could be completed for another £8,000, and it would then contain 60 beds.

The architect is Mr. Keith Young, and the contractors Messrs. Prestidge and Co.

## Figures and Tables

**Figure f1:**
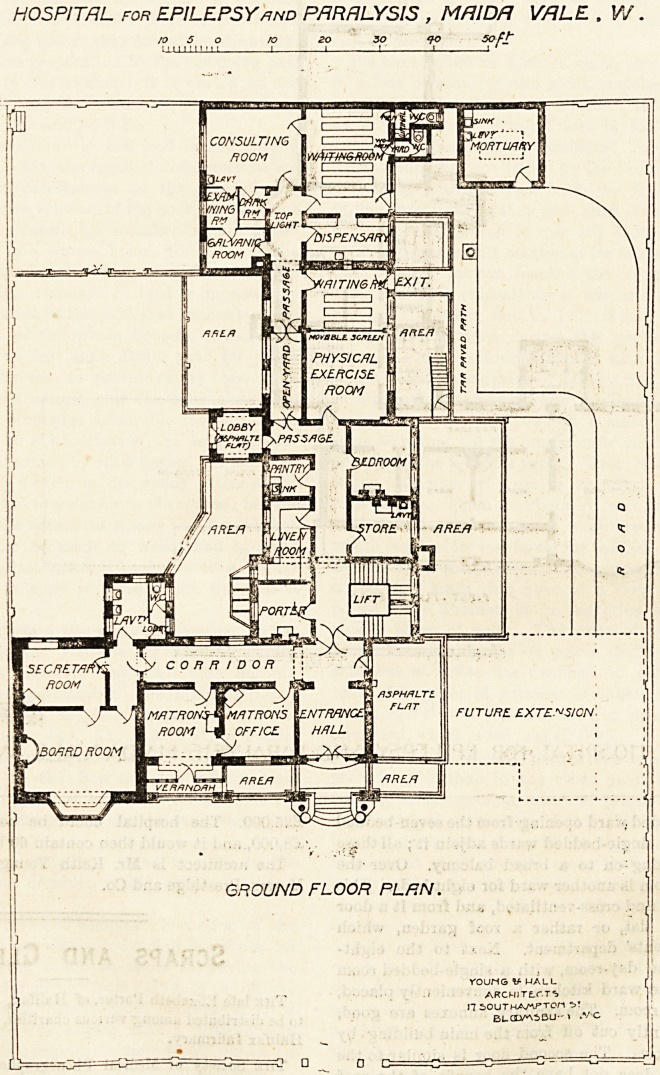


**Figure f2:**